# Flicker light stimulation enhances the emotional response to music: a comparison study to the effects of psychedelics

**DOI:** 10.3389/fpsyg.2024.1325499

**Published:** 2024-02-14

**Authors:** Caspar Montgomery, Ioanna Alicia Amaya, Timo Torsten Schmidt

**Affiliations:** ^1^Berlin School of Mind and Brain, Humboldt-Universität zu Berlin, Berlin, Germany; ^2^Charité – Universitätsmedizin Berlin, Einstein Center for Neurosciences, Berlin, Germany; ^3^Neurocomputation and Neuroimaging Unit, Freie Universität Berlin, Berlin, Germany

**Keywords:** flicker light stimulation, music-evoked emotion, visual hallucinations, psychedelics, stroboscopic light

## Abstract

Flicker light stimulation (FLS) is a non-pharmacological method of inducing altered states of consciousness (ASCs), producing hallucination-like phenomena as well as effects extending beyond the visual modality, including emotional effects. Research into the psychological and neural mechanisms of FLS is still in its infancy, but can be informed by research into other methods of inducing ASCs. For instance, research on classic psychedelics has reported enhancement of emotional responses to music. Here, we test to what degree FLS might also enhance the emotional response to music, using a study protocol designed to resemble a previous study on the effects of LSD as closely as possible, to allow for comparison of effect sizes across modalities and inform future research into FLS as an ASC-induction method. Twenty participants listened to emotionally evocative music in two conditions – with and without FLS – and reported on their emotional response to the music. FLS showed a significant enhancing effect on reported music-evoked emotion, especially emotions relating to “Joyful Activation”; additionally, we found that the experienced intensity of FLS correlated with reports of higher levels of emotional arousal. These findings motivate further research into FLS as a method for inducing ASCs and into the interactions between visual phenomena and music-evoked emotion.

## Introduction

Flicker light stimulation (FLS) is known to induce hallucination-like visual phenomena on demand (Rogers et al., 2020), where effects can extend to altered states of consciousness (ASCs) that are sometimes likened to hypnagogia (Bartossek et al., 2021). In typical usage of FLS as a relaxation- or meditation-supporting technique, a person reclines with their eyes closed while a light source with bright LEDs flickers at varying frequencies, typically in the range of 3–50 Hz. Despite the exposure to pure white light, participants typically report seeing colorful moving geometric patterns (Walter and Walter, 1949; Allefeld et al., 2011; Schwartzman et al., 2019), that are beyond volitional control (Ffytche, 2008), and vary with factors such as the rhythmicity and frequency of the FLS (Pearson et al., 2016; Amaya et al., 2023). Effects often go beyond the visual modality to include kinaesthetic, cutaneous and visceral sensations (Walter and Walter, 1949), as well as changes to mood, emotions, arousal and alterations to more abstract aspects of consciousness such as one’s sense of time (Walter and Walter, 1949; von Gizycki et al., 1998; Bartossek et al., 2021). Participants often report a positive state of relaxation that outlasts the flicker light exposure (Bartossek et al., 2021).

FLS is mostly used recreationally, but has also found applications in therapeutic contexts (Grof, 2019). Accounts of flicker-induced effects pre-date the scientific era [for instance, Nostradamus allegedly received visions by facing the sun and waving his hand in front of his closed eyes (ter Meulen et al., 2009)], and after being first formally documented by Purkyne (1819), the phenomenon began to be used in research and diagnostic purposes for epilepsy a century later (ter Meulen et al., 2009). FLS then started to be used as a recreational tool for exploring consciousness and in therapeutic or healing contexts in the 50’s and 60’s: “Dreamachines” using turntables to produce FLS were popularized (ter Meulen et al., 2009), while transpersonal psychologist Stanislav Grof has attributed his initial interest in psychiatry and ASCs to a session with a psychiatrist combining FLS with LSD (Grof, 2019). Since then, commercial FLS lamps, mobile phone apps and art installations [e.g., the modern-day “Dreamachine” project (Ranscombe, 2023)] have been developed, while FLS-like “strobe” lighting and similar visual stimuli have become a staple feature of dancefloors at nightclubs, concerts and festivals (experienced mostly, but not always, with eyes open). Within rave culture, these visual elements are often emphasized as important aspects in experiences of social connectedness and personal transformation through music-guided ASCs (Hutson, 2000; Sylvan, 2013; Newson et al., 2021; Mosina and Michael, 2023).

Music itself is a well-established tool for evoking and studying emotion (Krumhansl, 1997), including in the context of ASCs. Notably, Kaelen et al. (2015) found that music-evoked emotions – especially emotions linked to “Transcendence” – are enhanced by a high dose of the classic psychedelic LSD. Recent studies have begun to explore the corresponding neural correlates (Kaelen et al., 2016; Wall et al., 2023), including a focus on parahippocampal connectivity to visual areas (Kaelen et al., 2016). However, as with FLS, little is known about the psychological and neurological mechanisms of the emotional enhancement of music, beyond these preliminary findings. Accordingly, it is not clear how specific the enhancing effects are to psychedelics, or if other ASCs might also show comparable effects.

Answering how far the effects of enhanced emotional responses to music can also be effected by FLS (with its focus on the visual system) will cast some light on the nature of both the enhancement effect and the effects of FLS in general. Therefore, we asked whether and to what extent FLS enhances music-evoked emotion as previously reported for LSD. To find out, we conducted a comparison study to Kaelen et al. (2015), where we hypothesized that, compared to a control condition (exposure to music alone), an experimental condition of exposure to music plus FLS would enhance the emotional response to music. Furthermore, we expected this effect to correlate positively with the intensity of visual imagery experienced.

## Materials and methods

### Participants

Participants were recruited via mailing lists and word-of-mouth, meeting the following inclusion criteria as established by Bartossek et al. (2021): no history of epilepsy, migraines, or psychological problems (e.g., depression, anxiety disorders), and no current regular consumption of any psychotropic drugs (e.g., antidepressants, neuroleptics). To mitigate the risk of adverse events, we only included subjects who had either previously used FLS for recreational purposes, or been screened for indicators of photosensitive epilepsy with an EEG examination. Participants gave their written consent before commencing the experiment, filled out all questionnaires in English, and were paid €20 for participation. All materials and procedures were approved by the ethics committee at Freie Universität Berlin (application reference: 003/2023).

### Flicker light stimulation

FLS was delivered in a laboratory room at the Freie Universität, Berlin, using a CE-certified lamp system typically used in recreational applications (Lucia N°03, Light Attendance GmbH, Innsbruck, Austria). As in Kaelen et al. (2015), conditions of the room were adapted to promote feelings of comfort and safety, i.e., the lighting was dimmed and a blanket was provided.

To control for factors such as dynamics and changes to the stimulus, the lamp delivered constant, rhythmic flickering at a frequency of 10 Hz with a duty cycle of 0.3 (i.e., the bulbs were on 30% of the time). These stimulation parameters were not optimized for the most profound subjective experiences or the highest degree of immersion. Instead, they were chosen to be the same parameters as in previous controlled laboratory studies where subjective experiences were characterized in detail (Amaya et al., 2023) to enable verification that visual effects in the current setting are comparable to previously reported FLS effects.

The lamp was set to a default of 50% maximum brightness, emitting 3,500 Lumen. While this is less bright than in Amaya et al. (2023), pilot testing revealed that the light reaching the participants’ eyelids was similar when accounting for differences in distance to the light source [50 cm in this study compared to 150 cm in Amaya et al. (2023)]. Note that participants ultimately regulate the amount of light reaching the eye with the strength of squinting, a source of variability that is very difficult to control: we therefore sought to maximize comparability of stimulation between participants by optimizing the uniformity of the subjective intensity of the FLS. Participants were allowed to test the flicker brightness for a few seconds and to adjust it if necessary, with the guidance that the experience should be intense but not causing discomfort. Only one participant reduced the brightness by 10%.

### Music

Music was played via high-quality noise-canceling stereo headphones (Sennheiser Momentum 4), and the musical stimuli were identical to those used in the study of LSD by Kaelen et al. (2015). We followed the original study by testing the music volume for a few seconds and adjusting it if necessary, with the same criterion given to participants as for the FLS brightness (intense but not uncomfortable). This was essential for safety, and was designed to optimize the uniformity of the subjective intensity as much as possible (controlling somewhat for, e.g., different hearing acuity between individuals). Three participants reduced the volume by 15%. In all cases, the same levels of FLS brightness and music volume were used for the entire experimental session for each participant.

The playlists consisted of instrumental tracks (named in brackets with duration) from the following artists: Greg Haines (Playlist A: 183 Times, 09:08; Playlist B: Azure, 14:14), Brian McBride (Playlist A: Toil Theme Part 2 & Part 3, 05:11 and Mélodrames Télégraphiés Part 2, 04:12; Playlist B: Supposed Essay on the Piano, 4:10 and Mélodrames Télégraphiés Part 1, 05:25), Ólafur Arnalds (Playlist A: The Wait, 03:35; Playlist B: Autumn Day, 03:26), and Arve Henriksen (Playlist A: In the Light, 05:29; Playlist B: Leaf and Rock, 02:17).

The playlists are publicly available via Spotify (A: ^1^ B: ^2^), and were designed to be highly similar, but not identical: using subjective ratings, these two playlists had been tested and matched for emotional potency, liking and familiarity prior to the original study, i.e., the tracks were selected such that there were no significant differences between playlists on these measures (Kaelen et al., 2015).

The order of the playlists and the experimental conditions were both counterbalanced to control for possible confounding variables (25% of participants listened first to Playlist A with FLS, second to Playlist B without FLS; 25% listened first to Playlist A without FLS, second to Playlist B with FLS; 25% listened first to Playlist B with FLS, second to Playlist A without FLS; and 25% listened first to Playlist B without FLS, second to Playlist A with FLS).

### Questionnaires

Three questionnaires were used to assess personality traits potentially relevant to the measured outcomes, with all responses given as ordinal ratings on a five-point Likert scale. The Vividness of Visual Imagery Questionnaire (VVIQ) (Marks, 1973) and the Clarity of Auditory Imagery Scale (CAIS) (Willander and Baraldi, 2010) were used to measure “phantasia” – the ability to imagine perceptual phenomena – in the visual and auditory modalities, respectively, with each questionnaire containing 16 items. A subscale of ten questions from the 50-item International Personality Item Pool – Five Factor Model (IPIP-FFM-50) (Goldberg, 1999) was used to measure the personality trait openness.

With regards to the hypothesized emotional enhancement, the nine-item Geneva Emotional Music Scale (GEMS-9) (Zentner et al., 2008) was selected as the gold standard measure of music-evoked emotions that was used by Kaelen et al. (2015). Here, participants are asked to report the intensity with which they felt each of the emotions on a Likert scale from 1 (not at all) to 5 (very much).

Two questionnaires were used to assess participants’ visual experience during FLS and determine whether the prevalence of specific aspects were in line with previous studies. Participants rated 13 items from the five-dimensional Altered State of Consciousness Scale (ASC-R) (Dittrich, 1998), the most commonly used questionnaire in the assessment of altered states (Schmidt and Berkemeyer, 2018; Prugger et al., 2022), on a visual analog scale with “No, not more than usually” and “Yes, much more than usually” as anchors. These items were: the subscales for simple imagery (items 14, 22, and 33), excluding 33 (“I saw brightness or flashes of light with closed eyes or in complete darkness”) since it is an intrinsic aspect of the FLS experience; complex imagery (items 39, 72, and 82); and audio-visual synesthesia (items 20, 23, and 75); and, following Amaya et al. (2023), five other individual items of relevance to the FLS experience (“My sense of space and time was altered as if I was dreaming,” “The boundaries between myself and my surroundings seemed to blur,” “I felt as if in a wonderful other world,” “I had the impression I was out of my body,” and “I felt sleepy”). Five questions were taken from the newly-developed Stroboscopic Visual Experience Survey (SVES) (Amaya et al., 2023) to measure the extent to which participants saw “Klüver forms” and motion in their visual experience, with “Yes” and “No” forming the anchors for the visual analog scale. (Klüver forms are a set of “form constants” which consistently show up in altered vision, namely: tunnels, spirals, cobweb-like targets, and grids, i.e., lattices including honeycombs, checkerboards, and triangles. See (Klüver, 1966) for more information.)

Finally, to test how far FLS (with music) produces other alterations to consciousness beyond visual effects, and make further comparisons to psychedelics, we used a further 29-item visual analog scale from Kaelen et al. (2015), where “No, not more than usually” and “Yes, much more than usually” were the anchors. This questionnaire, the GEMS-9 and two further questions asked verbally during the experimental sessions (see below) were used identically in both studies, and therefore allow for direct comparison of our results to the effects of LSD. The SVES was included to measure subjective visual experiences more precisely, the ASC-R as a canonical questionnaire for capturing widespread alterations to consciousness. These two questionnaires allowed for comparison of the FLS-with-music experience to previous FLS literature (Bartossek et al., 2021; Amaya et al., 2023).

### Experimental procedure

A semi-structured interview took place before the day of testing to screen participants for eligibility. Upon arrival for testing, participants were briefed on the study and the potential effects of FLS before signing the consent form. Thereafter, participants provided basic information about demographics and previous experiences with psychedelic substances, and completed the VVIQ, CAIS, and IPIP-FFM questionnaires via tablet.

Lying on a comfortable reclining chair with the lamp 50 cm above their face, each participant was instructed to close their eyes and keep them closed for the duration of each playlist. After testing the brightness and volume and adjusting if necessary, Playlist A or B was played with or without the accompaniment of FLS, depending on the counterbalanced order of the conditions and playlists. After each track, the experimenter triggered a pre-discussed prompt for the participant to remove the headphones by tapping them on the shoulder. Two verbal ratings regarding the experience during the previous track were then collected: “How emotionally affected were you by the music, from 0 (“not at all”) to 100 (“very much”)?” and “Please rate the subjective intensity of the effects of the flicker-light on a scale of 0 (“no effects”) to 10 (“extremely intense effects”).” This was the same procedure followed by Kaelen et al. (2015). FLS was stopped after the last track of the playlist, and participants were told to open their eyes, remove the headphones, and ground themselves back in the room, before completing the questionnaires via tablet. After a short break, this procedure was repeated for the second condition.

### Data analysis

All statistical analysis was conducted using Rstudio (v1.4.1103), closely following, and extending, the procedures used by Kaelen et al. (2015). Responses for all visual analog scales were converted into scores from 0 to 100 for analysis. To compare between the two conditions, paired t-tests were used wherever data from the questionnaires were normally distributed (determined with Shapiro–Wilk normality tests); otherwise the Wilcoxon sign-rank test was performed to confirm the significance results of the t-tests (which are reported for comparability reasons). Following Kaelen et al. (2015), this includes ratings for the GEMS-9, despite this being ordinal data. We applied false discovery rate (FDR) correction to control for alpha-error inflation in multiple comparisons (Benjamini and Hochberg, 1995).

Pearson correlation coefficients were calculated to evaluate hypothesized relationships between the average subjective intensity of the FLS and (i) the intensity of emotional arousal in response to music under FLS (i.e., the average score to the question “How emotionally affected were you by the music?”), and (ii) its enhancement by FLS (i.e., the average difference in answers to the question “How emotionally affected were you by the music?” between conditions). Further correlational analyses were performed to explore: (i) possible effects of personality traits (openness or phantasia) or prior psychedelic experience on overall emotional arousal, and (ii) any aspects of the FLS experience (simple and complex imagery, synaesthesia, Klüver forms) that correlated with emotional arousal in the FLS condition. FDR correction for multiple comparisons was again applied for all correlation statistics.

## Results

### Participant demographics

N = 20 healthy volunteers completed the study [13 female, 7 male; age = 27.5 ± 4.7 (*M* ± SD), 22–39 years (range); 4 left-handed]. Personality trait scores were as follows: openness = 41 ± 4.1, 28–47; phantasia (visual) = 61 ± 9.3, 46–75; phantasia (audio) = 67 ± 10.3, 46–80, indicating our sample to be within a normal range, not displaying a specific sampling bias (Constantinescu and Constantinescu, 2016; Dance et al., 2021; Floridou et al., 2022). 18 of our participants had at least one previous experience with classic psychedelics (mean experiences = 17 ± 20, range = 2–70; days since last experience = 430 ± 566, 6–1,650).

### Effects of FLS on the emotional response to music

**Figure 1:**
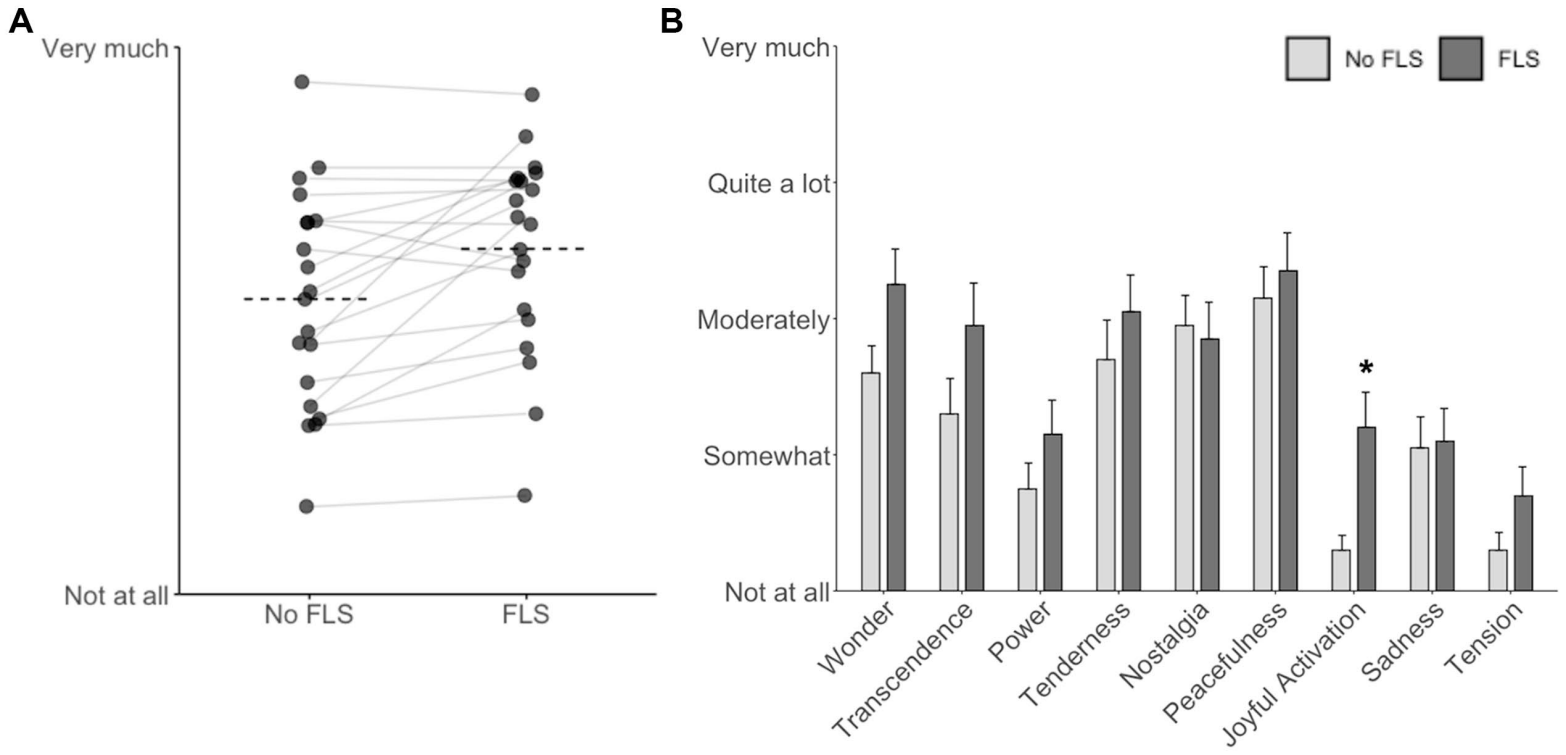
Effect of FLS on music-evoked emotions. **(A)** The primary outcome of the experiment: each data point represents one participant’s average response to the question “How emotionally affected were you by the music?” The dashed horizontal line represents the group average for each condition, showing significantly higher ratings under FLS than in the control condition. **(B)** Emotional effects assessed by the GEMS-9 show increased responses for all items except “Nostalgia”. Scores were significantly higher with FLS than the control condition for “Joyful Activation”. Bars represent mean scores plus standard error of the mean (SEM); *p < 0.05 after FDR correction.

For the primary outcome of the experiment, paired t-tests identified a significant effect of FLS on the emotional response to the presented music (Cohen’s *d* = 0.740, *t*(19) = 3.310, *p* = 0.004). This reflected that mean scores (between 0 and 1) for all music stimuli to the question “How emotionally affected were you by the music?” were significantly higher for the FLS condition (M ± SD = 0.63 ± 0.18) than for the control (0.54 ± 0.20; Figure 1A). The effect was independent of both the order of the conditions (*t*(19) = 0.603, *p* = 0.554), and which playlist accompanied the FLS (*t*(19) = 1.165, *p* = 0.259).

Furthermore, for each emotion on the GEMS-9 except “Nostalgia”, mean ratings were higher with FLS than in the control condition (Figure 1B). Of these, the difference was significant for “Joyful Activation” (*t*(19) = 3.758, *p* = 0.001), “Wonder” (*t*(19) = 2.668, *p* = 0.015), and “Transcendence” (*t*(19) = 2.096, *p* = 0.05), but significance survived FDR correction for nine simultaneous comparisons only for “Joyful Activation”. This difference was also significant when non-parametric Wilcoxon sign-rank tests were applied.

### Subjective effects of FLS

**Figure 2:**
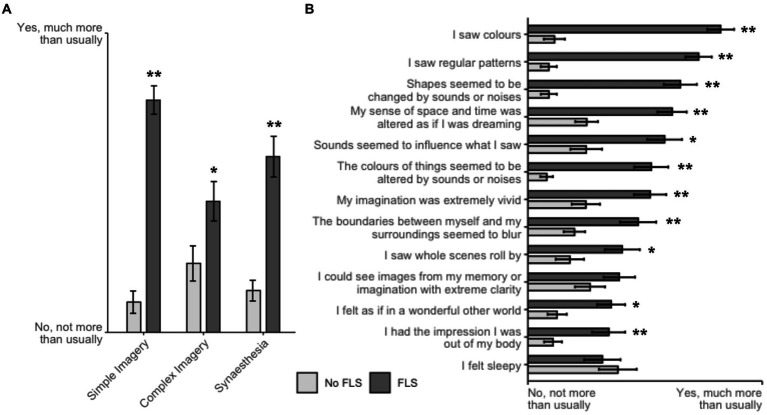
Visual effects of FLS. Visual and other experiential changes with FLS as measured by subscales **(A)** and items **(B)** of the ASC-R. Bars represent the mean and SEM; *p < 0.05, **p < 0.001 after FDR correction for multiple comparisons.

To verify whether participants experienced typical FLS effects as described in previous studies, and to quantify potential demand characteristics, we tested the effects of FLS on ASC-R and SVES questionnaire items and found that the FLS condition produced a notably different visual experience compared to the control condition. In the ASC-R questionnaire, after FDR correction for multiple comparisons and confirmation with non-parametric Wilcoxon sign-rank tests, ratings for all three subscales (Figure 2A) and 11 of the 13 individual items (Figure 2B) were significantly increased by FLS (*p* < 0.05 FDR corrected for three and 13 tests, respectively). The SVES revealed much higher prevalence of Klüver forms [*t*(18) = 14.045, *p* < 0.001; FLS: 72.2 ± 17.3 (*M* ± SD), No FLS: 9.4 ± 14.1] and motion [*t*(18) = 7.443, *p* < 0.001; FLS: 91.8 ± 18.5, No FLS: 26.1 ± 36.7] in the visual field, effects which again were significant after FDR correction for two comparisons. One participant’s ratings were excluded because they mistakenly thought that binary answers were required instead of a continuous scale.

**Figure 3:**
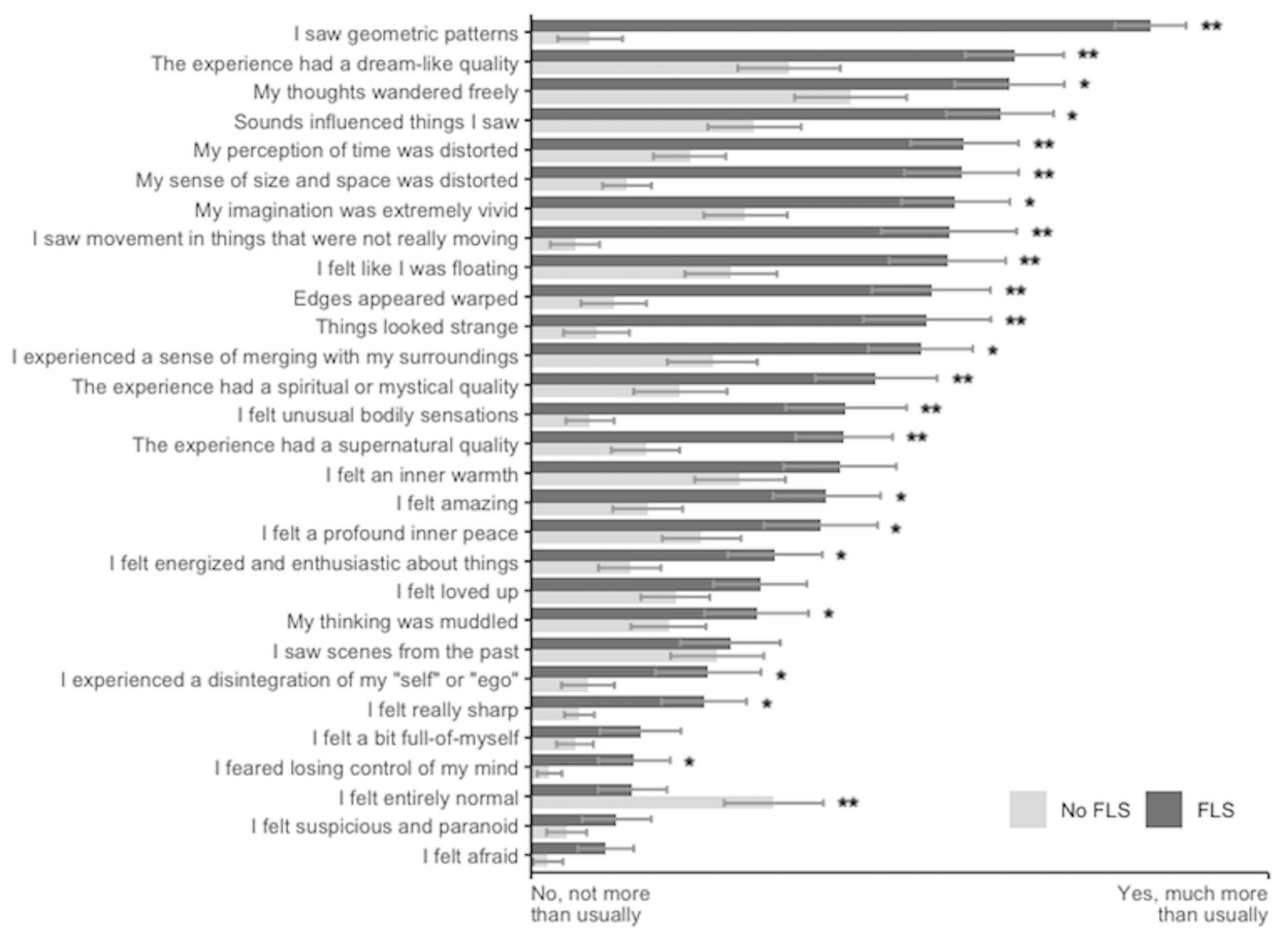
Altered state of consciousness induced by FLS with music. FLS significantly increased ratings for 22 of the 29 items used in the visual analog scale. Bars represent the mean and SEM; *p < 0.05, **p < 0.001 after FDR correction for multiple comparisons. The same questionnaire items as the original study were included [see Supplementary material in Kaelen et al. (2015)] to enable a direct comparison to LSD, including items relating to vision. Since our participants had their eyes closed, the comparability to other studies where these items relate to open-eye visual experiences is limited.

Finally, we tested FLS effects against a 29-item visual analog scale to see how far FLS produces an altered state of consciousness, beyond visual and emotional changes. We found that 22 of the items showed significant increases in the FLS condition (*p* < 0.05 FDR corrected for 29 items; Figure 3). We tested intra-rater reliability with the item “My imagination was extremely vivid,” which appeared on both the ASC-R and the 29-item visual analog scale questionnaires: for 90% of participants, responses were within 20 percentage points of each other across the two questionnaires.

### Correlation analyses

In the FLS condition, as hypothesized there was a significant and moderately strong positive correlation between the average subjective intensity of the flicker light and the average emotional arousal by the music (*r* = 0.529, *p* < 0.05 FDR corrected for two tests). Furthermore, the level of FLS-induced complex imagery (as a subscale of the ASC-R) also positively correlated with emotional arousal (*r* = 0.457, *p* = 0.043), although this was not significant after FDR correction for three comparisons. Contrary to our expectations, we saw no indication that previous psychedelic use, phantasia, or openness had any bearing on the intensity of the FLS experience, music-evoked emotional arousal, or its enhancement by FLS.

## Discussion

In this experiment, we closely followed the protocol from a previous study to investigate the enhancement of music-evoked emotion in altered states of consciousness induced by flicker light stimulation. We exposed 20 participants to two playlists of instrumental music, both with and without rhythmic 10 Hz FLS as an accompanying stimulus, and collected ratings of music-evoked emotion, as well as other aspects of the experience. We found that FLS significantly increased participants’ emotional response to the music, especially emotions related to “Joyful Activation”. Furthermore, emotional arousal in the music-with-FLS condition positively correlated with perceived intensity of the FLS.

### Visual FLS effects and their interaction with emotional responses

As expected, the questionnaires revealed large alterations in participants’ visual experience when exposed to FLS, especially with regards to colors, typical Klüver patterns, movement, and synesthetic phenomena. This demonstrates typical FLS effects as seen in previous studies. However, the alterations to consciousness were reported as more severe than in a previous study which did not use music (Amaya et al., 2023): the SVES showed higher ratings of Klüver forms (*M* ± SD = 72 ± 17.3 versus 59 ± 36.5) and motion (92 ± 18.5 versus 80 ± 24.5) in the visual experience, and all items on the ASC-R were also scored higher (e.g., “I saw regular patterns”: 73 ± 25.0 versus 47 ± 32.6; “My sense of space and time was altered as if I was dreaming”: 62 ± 25.1 versus 24 ± 29.2). This suggests that the addition of music to FLS produces a more radically altered state of consciousness than FLS alone (although other paradigm differences may have affected effect sizes).

Our correlation analyses support the notion that emotional responses to music under FLS may be associated with the visual phenomena induced by FLS. Not all participants responded to FLS with enhanced emotionality (see Figure 1), and the strongest correlate with emotional arousal under FLS-with-music was the intensity of their visual FLS experience. There was a suggestion that the more abstract and meaningful visual phenomena in complex imagery may be linked to increased emotion. More speculatively, the aforementioned increase in Klüver forms and motion when music is added to FLS may arise from a bidirectional influence between FLS-induced visuals and music-evoked emotion, such that increasing one amplifies the other.

### Further phenomenal effects of FLS

The 29-item questionnaire exploring wider subjective effects of FLS showed widespread alterations to consciousness, which were nevertheless most pronounced in the items most related to vision. For instance, “I saw geometric patterns” scored highest by far, surpassing even corresponding ratings for the psychedelics LSD (Kaelen et al., 2015) and psilocybin (Muthukumaraswamy et al., 2013) from studies using the same measures, along with items relating to audio-visual synesthesia and autobiographical imagery. Interestingly, scores for distortions of time and space, and the “dreamlike,” floating, and thought-wandering qualities of FLS were also higher than have been found for LSD and psilocybin; conversely, profound changes in affect – feeling “amazing,” “an inner warmth,” “energized and enthusiastic,” “a profound inner peace,” “sharp,” and “loved up” – seem to be more pronounced with psychedelics, perhaps corroborating the notion that FLS experiences are closer to hypnagogia than psychedelic trips. Ego-dissolution, merging with surroundings, unusual physical sensations, and mystical, spiritual and supernatural features of FLS were somewhat less prevalent, but still significantly increased compared to the control condition, while negative effects (fear, suspicion and paranoia) were largely absent. In sum, the results demonstrate that FLS alters consciousness beyond visual and emotional domains.

### Enhancement of emotion by FLS compared to LSD

Importantly for a comparison of the emotion-enhancing effects of FLS and LSD, baseline ratings from our control condition were extremely similar to those from the placebo condition in Kaelen et al. (2015) with regards to music-evoked emotional arousal and the GEMS-9. This is reassuring, since it indicates that additional effects of “set and setting” (Hartogsohn, 2017), differences between the groups of participants and in demand characteristics are all unlikely to be relevant in the given comparison. Participants in the current study were higher in openness (Gow et al., 2005) and had much less prior psychedelic experience than the participants in the LSD study (Kaelen et al., 2015) but more experience than the general population (Winstock et al., 2021). However, these factors are unlikely to have affected the results as we did not find evidence in the correlation analyses that psychedelic experience, openness or phantasia would have had an effect on the outcomes measured. Furthermore, whereas we randomized and controlled for effects of the order of the conditions, all participants in the original study had the LSD condition second, which may have influenced effect sizes.

Whereas LSD increased average emotional arousal scores from 0.51 to 0.71 (Kaelen et al., 2015), FLS increased ratings by roughly half as much, namely from 0.54 to 0.63, with comparable distribution of the data. This represents a moderate-to-large effect size (Cohen’s *d* = 0.74). With one exception (“Nostalgia”), FLS resembled LSD effects in showing higher average scores for all music-evoked emotions in the GEMS-9 compared to the control condition. In fact, the overall resemblance between these ratings in the two studies is striking [compare Figure 2 with Figure 2 in Kaelen et al. (2015)]: the pattern of responses was the same, with lower strength of effects in the present study. Thus, the increases in “Transcendence”, “Wonder”, “Power”, and “Tenderness” exceeded significance thresholding after FDR correction with LSD, but not FLS.

### Limitations

This study used a relatively small sample size, as is typical for studies stepping into unexplored areas of research (Shen et al., 2011; Szucs and Ioannidis, 2020). For instance, our protocol was informed by two studies on the effects of the frequency and rhythmicity of FLS (Amaya et al., 2023), and the neural correlates of the FLS-induced state (Schwartzman et al., 2019), which used 20 participants and 23 participants, respectively. Similarly, two previous studies on visual phenomena in psychedelic-induced states (de Araujo et al., 2012; Kaelen et al., 2015) reported findings from only 10 participants. Studies of this kind are clearly limited with regards to their generalizability, but still valuable in informing future research.

One question for future research is to test how different types of music will resonate with different participants and what type of music will lead to strongest effects. For the current comparison, a narrow range of music was tested, and it remains an open question how far the findings can be generalised across all genres. In fact, anecdotal comments from participants suggested that the fast, intense, rhythmic FLS was somewhat incongruous with the slow, spacious, melodic music – presumably for the same reason that strobe lighting is found in electronic dance clubs instead of acoustic concert halls. This relates to another point, which is that we set the FLS settings to 10 Hz rhythmic flickering without variation. This was good for maximizing experimental control and comparability to previous studies, but not optimal for producing the strongest ASC effects. We therefore speculate that effect sizes could have been larger – and subjective experiences more interesting – with customized flicker dynamics and music better suited to the FLS experience.

### Outlook

The results of the study contribute to ongoing consciousness research. Our main finding that FLS enhances the emotional response to music raises the possibility that visual phenomena drive other aspects of (some) ASCs. For instance, it may be that immersion in colorful patterns, geometries and complex imagery invites people deeper into the experience and helps them to “let go” and surrender to emerging emotions, such as those that arise in relation to music. Alternatively, FLS may just heighten awareness of emotions that are present anyway; either way, visual phenomena are implicated in the induction of emotional experiences in a manner that demands further attention, especially in light of indications from the latest literature that the quality of one’s visual experience under psychedelics may bear upon therapeutic outcomes (Aaronson et al., 2023; Aqil and Roseman, 2023).

Furthermore, based on our findings, we cautiously speculate that the relation between music-evoked emotional arousal and visual phenomena in ASCs may be synergistic and bidirectional. This idea is assumed by a mode of therapy called the Bonny Method of Guided Imagery and Music (BMGIM), inspired by extensive work with ASCs in the 1970s (Bonny and Pahnke, 1972), which uses music to evoke and explore mental imagery to promote well-being and exploration of consciousness in participants, with apparent success (McKinney and Honig, 2017). It is also perhaps consistent with a wide range of long-standing shamanic practices that use music to enhance visual experiences, and vice versa. For instance, in traditional Amazonian settings, quasi-synaesthetic “visions” shaped by emotionally powerful songs (*icaros*) have long been considered a main vehicle for the effects of ceremonial ayahuasca use (Luna, 1992; Shanon, 2002; de Araujo et al., 2012; Ruffell et al., 2023).

Finally, while FLS is not the same as psychotherapy, nor as an experience with classic psychedelics, it could serve as a model for certain aspects of both in order to address open questions in ongoing research, as psychedelic-assisted therapy is increasingly studied for its therapeutic potential (Carhart-Harris and Goodwin, 2017; Nutt et al., 2023). For instance, there is uncertainty as to which features of the experience (if any) are important for therapeutic outcomes, and (therefore) how they can be leveraged optimally. While it would be a stretch to suggest that visual effects alone could be of major therapeutic value, the emotion-enhancing effects of FLS may prove valuable for some therapeutic approaches. One potential application of FLS could be in preparation for psychedelic experiences, or during the crucial period of integration following a psychedelic session (Watts and Luoma, 2020; Bathje et al., 2022). For instance, anecdotal reports suggest that relistening to the music used in dosing sessions is a valuable part of the integration process: these benefits could conceivably be enhanced by combining the music with FLS. In sum, the use of FLS to enhance emotional responses to music may have applications beyond recreational applications; however, this remains speculative until further systematic testing occurs.

## Conclusion

The study at hand provides evidence that FLS enhances the emotional response to music. The intensity of the visual experience induced by FLS seems to be a significant driver of this effect, and emotions related to “Joyful Activation” are particularly affected. The enhancement effect seems to occur in a manner similar to with classic psychedelics, albeit to a lesser extent. This effect indicates a possible role for visual effects in the mechanism of emotion enhancement in ASCs, motivating further research into the use of FLS (with music) which may have hypothetical value for informing therapeutic approaches. Further research is needed to test whether these findings hold for wider populations and other genres of music, and whether visual phenomena and emotional arousal are mutually reinforcing aspects of ASCs.

## Data availability statement

Data are available on the OSF data repository (URL: https://osf.io/hnqc5). Further inquiries can be directed to the corresponding author.

## Ethics statement

The studies involving humans were approved by the Ethics Committee at Freie Universität Berlin. The studies were conducted in accordance with the local legislation and institutional requirements. The participants provided their written informed consent to participate in this study.

## Author contributions

CM: Conceptualization, Data curation, Formal analysis, Investigation, Methodology, Visualization, Writing – original draft, Writing – review & editing. IA: Conceptualization, Formal analysis, Methodology, Software, Writing – review & editing. TS: Conceptualization, Funding acquisition, Methodology, Project administration, Supervision, Writing – review & editing.

## Footnotes

^1^ https://open.spotify.com/playlist/2dWd3GUUSmkGMI8PjOAxjk

^2^ https://open.spotify.com/playlist/5eB12ZQInHR08N1o3d3bzV
